# Precarious work and methodological challenges to study hard-to-reach populations

**DOI:** 10.11606/s1518-8787.2024058005470

**Published:** 2024-03-27

**Authors:** Rita de Cássia P. Fernandes, Janaína Santos de Siqueira, Matheus F dos Santos, Paulo G. L. Pena, Guilherme L. Werneck, Alex Burdorf

**Affiliations:** I Universidade Federal da Bahia Faculdade de Medicina da Bahia Salvador BA Brasil Universidade Federal da Bahia. Faculdade de Medicina da Bahia. Salvador, BA, Brasil; II Universidade Federal da Bahia Programa de Pós-graduação em Saúde, Ambiente e Trabalho Salvador BA Brasil Universidade Federal da Bahia. Programa de Pós-graduação em Saúde, Ambiente e Trabalho. Salvador, BA, Brasil; III Universidade Federal da Bahia Programa de Pós-graduação em Saúde Coletiva Salvador BA Brasil Universidade Federal da Bahia. Programa de Pós-graduação em Saúde Coletiva. Salvador, BA, Brasil; IV Universidade Federal do Rio de Janeiro Instituto de Estudos em Saúde Coletiva Rio de Janeiro RJ Brasil Universidade Federal do Rio de Janeiro. Instituto de Estudos em Saúde Coletiva. Rio de Janeiro, RJ, Brasil; V University Medical Center - Erasmus MC Department of Public Health Rotterdam The Netherlands University Medical Center - Erasmus MC. Department of Public Health. Rotterdam, The Netherlands

**Keywords:** Epidemiology, Social Vulnerability, Occupational Groups, Surveys and Questionnaires, Methodology as a Subject

## Abstract

**OBJECTIVE:**

To describe the methodological challenges and strategies of a web survey on the working conditions and health among delivery workers.

**METHODS:**

The study population consisted of Brazilian delivery workers operating in the national territory. Procedures include building solid and ongoing collaboration with worker representatives and conducting a four-month data collection from February to May 2022, sharing the link to the online questionnaire on social media such as social networks (Facebook, Instagram) and messaging apps (WhatsApp, Telegram).

**RESULTS:**

The recruitment of 41 leaders or influencers of delivery workers increased the dissemination of the study, some of whom participated in the consensual validation of the questionnaire; the production of content for social media for the dissemination of the questionnaire link on social networks and applications, and the in-person dissemination of the study at the delivery workers’ meeting points during the workday played a fundamental role, totaling around 132 hours in 45 shifts. The strategies adopted for data collection with a hybrid approach to dissemination made it possible to carry out the web survey. After four months of the web survey, 564 delivery workers, 543 men and 18 women, responded to the online questionnaire.

**CONCLUSION:**

The web survey presented methodological strategies to overcome the challenge of reaching workers, including hybrid work, to increase the participation of workers, on whom epidemiological research is still scarce.

## INTRODUCTION

Precarious work has characterized the inclusion of a large portion of workers in the labor market. The dimensions of precarious work imply greater flexibility in labor relationships and, consequently, erosion of working conditions. Unstable relationships characterize precarious work in its multidimensionality, with temporary contracting, involuntary part-time work, or outsourcing; inconsistent income; and insufficient rights and social protection, weakening the collective representation of workers, implying a low power to oppose degrading working conditions^1,2^.

In this scenario, both the psychosocial demands at work and the physical and cognitive demands must increase. Nonetheless, most of the evidence on the effects of strenuous working conditions on a person’s health derives from studies with occupational groups that were accessible to researchers. Many vulnerable groups, however, are more elusive to researchers—such is the case with the workers of the gig economy.

Accessing data on work-related health problems under these conditions is a difficult task, given the dispersion of this workforce, with no stable employment relationships, subject to unregulated working hours, whose determinant of continuity is the daily income to guarantee survival^3^. Therefore, it is a population difficult to reach. This gives rise to the paradoxical situation that those most at risk of adverse working conditions are the least studied.

Under these conditions, there is no common register that allows estimating the population in the different professional categories and monitoring it on a territorial basis, enabling the adoption of a conventional sampling strategy^4^. This is the situation of workers subordinated to the platform economy, particularly those who provide services on the streets, such as delivery workers (DW).

The challenges for epidemiologic studies with vulnerable social groups are discussed in the public health literature, highlighting in this perspective the so-called “hidden populations” or hard-to-reach groups. The challenges faced by researchers in accessing these populations have different motivations. Still, the fact that they are socially vulnerable groups is a common issue^5^. The difficulties of extensive, population-based research addressed in this article resulted in methodological developments that emphasize the recruitment process of these populations and the central role of social relationship networks^6,7^.

Besides the difficulty of accessing workers living in precarious conditions among vulnerable social groups, once they are reached, the challenge is to make them participate in a research initiative whose results will not return in immediate benefits. The time to respond to an instrument will compete, given the scarcity of time, with the delivery company’s requirement for availability during working hours.

The epidemiological literature on the health of workers subordinated to digital platforms, particularly delivery workers, is still scarce, with studies involving few participants. The difficulty of accessing these vulnerable workers is a strong explanatory hypothesis for the reduced epidemiological literature, considering the paradox that this difficulty represents, given the significant volume of data available on digital platforms, a field where researchers and surveillance services in workers’ health did not have access^8^. In 2002, a study with bicycle delivery workers reached 113 workers in a survey that investigated accidents at work^9^. As of 2014, studies were found that addressed traffic accidents with secondary data from national information systems, hospital records, and insurance compensation systems, involving delivery workers in countries such as South Korea and Australia^10^. Two recent studies with primary data addressed the association of job burnout, sociodemographic, occupational, and psychosocial characteristics with aspects related to traffic security, involving 434 and 553 delivery workers in Athens and Vietnamese cities, respectively^13,14^. Furthermore, an action-research report provided results on the working conditions of 500 delivery workers in New York City in 2021^15^: “App-delivery workers are exposed to risk of severe injuries, which can prevent them from working for several weeks, without health insurance or financial cushion. Forty-nine percent of survey respondents reported having been in an accident or crash while doing a delivery.”

Therefore, strategies are required to overcome barriers and expand access, enabling epidemiological research and obtaining primary data on this occupational category.

Web surveys have become one of the main alternatives for collecting primary data in the COVID-19 pandemic scenario, given the need for social distancing and the advantage of the short period in which, in principle, it would be possible to obtain results^16^. However, despite its expressive potential reach, numerous challenges surround this type of epidemiological research, especially when seeking to investigate specific groups in situations of relevant social vulnerability, as is the case with DW of goods.

This study aims to describe the course of this epidemiological research, addressing the techniques and procedures adopted for conducting a web survey among workers who deliver goods via digital platforms.

## METHODS

### Study Design and Population

A cross-sectional study, typified as a web survey, was carried out. The study population consisted of workers—men and women—who deal with the delivery of goods, work in national territory, and consented to participate in the research via a free and informed consent form.

### Survey Content Development

Since the final planning period of the survey, from September 2021 to January 2022, the procedures were focused on in-person and online (social media) activities, aimed at mapping DW on national and local WhatsApp groups, obtaining cell phone numbers, mapping areas where DW gather during working hours, identifying leaders or influencers in the category (LICs), creating profiles for the study on Instagram and Facebook.

Identifying these LICs was one of the stages for recruiting participants. This procedure is somewhat similar to that adopted in studies guided by Web-based Respondent Driven Sampling^4^. Respondent-driven sampling, proposed in the 1990s by Heckathorn^7^, aimed at developing techniques to approach what the author called “hidden populations,” characterized by not having a framework for sampling; since they are strongly concerned with privacy, which determines high rates of refusal and the possibility of information biases due to the fear of being identified, population size and population boundaries are unknown. Since the first studies, other developments have proposed to expand the range of investigated populations, including “hard-to-reach” groups, such as workers living in precarious conditions^4^. What remains as the basis of this technique, and which is a reference in this web survey, is that participants recruited from the target population are invited to participate in the study and trigger participation in a network of respondents, in a flow that is progressively expanded until the research is completed^4,5,17^.

LICs were sought from all regions of the country. The identification and recruitment of these leaders continued during the online data collection, with new recruitments until its closure. The research team shared the study’s objectives and the strategies for its execution with these workers, after hearing their perception about the opportunity and relevance of the research. With this connection, the LIC became potential interlocutors for each new strategy to increase the number of respondents via their social media and “live broadcasts” with synchronous and asynchronous audiences of DW.

### Data Capture Methods and Activities

In the planning phase, possible scenarios were collectively constructed to reach DW. Consensual validation of the research questionnaire was carried out, with verification of the language used in formulating the questions to bring it closer to the common language adopted by the category, with the participation of four LICs with known experience as DW in different territories of the country (Rondônia, Distrito Federal, Bahia, and Pernambuco).

Adaptation by repeatedly revising the research instrument to obtain a concise format, with questions in accessible language, culturally oriented, and with response scales that allowed faster understanding by the DW.

The need for a questionnaire that allowed for less time to respond required difficult choices. These choices were made after assessing what would be essential to obtain data for this study, since the research team identified no other epidemiological research with DW in the country. Based on a digital platform, the research instrument was presented to the target population via a link sent on social media—messaging applications WhatsApp and Telegram, in groups or individually, and social networks Instagram and Facebook (delivery workers’ profiles and groups).

The very different strategies of this study, for example, the personal approach, which requires the respondent’s cell phone number, and the group approach, which entails standard web pages visited and a continuous search for them, based on the social media of the DW category, are the results described below.

This study is part of a larger project approved by the Research Ethics Committee, under registration number 5.258.142.

## RESULTS

The mapping of DW in social media was carried out by an active search on YouTube and social networks, using hashtags, and by checking the interaction of DW on the profiles of leaders or influencers of the category or profiles directed to this category (e.g.: motorcycle sales and maintenance services).

Forty one DW were identified and recruited, characterized in Table 1, who played the role of LIC to disseminate the web survey and increase the number of respondents. There were many interactions between the research team and each of the LIC, and some illustrate the process. During the adaptation of the language of the questionnaire to the DW community, for example, the measurement of “low back pain” gave way to “pain in the spine,” according to recognized morbidity and customary use in the community, for pain in the lower back. In research in the world of work, information biases may play a relevant role, given the possibility that the worker may fear the lack of confidentiality of the information provided. In addition to the anonymous questionnaire, the first variable, for example, the date of birth, was reduced to the age-related variable: “because the guys are a little brooding,” according to one leader, during the consensual validation phase.

**Table 1:** Characteristics of category leaders or influencers recruited for the EpisSAT Entregadores web survey.

Characteristics of delivery workers recruited as leaders or influencers in the category	Amount	Brazilian states Brazilian federation
A president of a union organization at national level	1	
Presidents of union organizations at state or local level	6	BA, DF, PE, RS, SP
Other board members of union organizations	4	BA, MT, PE
Presidents or members of category associations	10	BA, CE, DF, RO, MS, SP, RJ
Delivery workers in social collectives or with some performance in social movements related to the labor rights of the category	12	BA, ES, GO, MG, RJ, RR, SP
Delivery workers who are influencers on social media—Instagram and YouTube	8	AL, MG, PI, RS, PR, SP
Total of recruited leaders or influencers	**41**	

A total of 134 active groups of DW were identified on WhatsApp, Facebook, and Telegram. The administrators of these social media groups were informed about the objectives of the study before disseminating the link and were consulted about the possibility of periodically disseminating this web survey.

Insertion into WhatsApp groups made it possible to extract more than 5,700 cell phone numbers from this messaging application, which were stored in an Excel spreadsheet. Software was used to extract these contacts in a short period for free. Subsequently, the work had to be conducted manually.

The questionnaire link was sent mainly on WhatsApp; on Telegram, it was accompanied by standardized texts, with language adapted and renewed each week of collection, with an invitation to participate in the study.

Phone calls were made when communication with the DW required it, such as when insecurity regarding the veracity of the research and the validity of the link was revealed. Reports of “fake surveys” experienced by the category, sponsored by the owners of app-delivery businesses and denounced by investigative journalism alerted the team to look for new and better communication strategies and to assure DW of the origin of the research at the federal public university^18^.

Profiles were created and managed on the survey’s social media to disseminate the study and communicate with DW, adopting community-oriented language in the digital environment. The Instagram profile (@epissat_entregadores) acted as the project’s headquarters, where it was possible to verify the veracity of the link and access the content produced. On this network alone, 57 publications were made (in the feed), which included the official video of the study (Link), as well as videos with the research team at the public university and with DW encouraging the participation of their peers and referring to potential contributions of the survey.

A summary of the data collection activities, stratified by virtual and in-person actions, can be found in Table 2.

**Table 2:** Data collection activities, stratified by virtual and in-person actions, for the EpisSAT Entregadores web survey.

Actions in the virtual environment, sorted by date, since the beginning of the study
Disclosure in live broadcast by a Southern LIC on YouTube
Two videos of LICs supporting the study
Disclosure in live broadcast by a LIC
Promotion of a “clickable” card
Disclosure to personal cell phones via messaging apps
Disclosure in live broadcast by a Southeast LIC on YouTube
Disclosure on the websites of the largest DW union in Brazil (SP)
Dissemination by a newspaper at the state level (online)
Researcher interview at a state radio station
Article on a public university website and its dissemination on social networks
Article on national television news and its dissemination on social networks
Dissemination by one of the researchers in a Southern live broadcast on YouTube
Instagram post boost
Video with many LICs supporting the study
Video of a researcher with informative content
Second video with many LICs supporting the study
Face-to-face actions and articulation with LICs, sorted by date, since the beginning of the study
Meeting to discuss the objectives and procedures of the study with LICs from four different states
Beginning of in-person dissemination of the study at DW meeting points in one capital of a Northeastern state
Start of obtaining personal cell phone number at DW meeting points
Dissemination by a LIC with national operations
Start of support from the president of the largest DW union in Brazil (SP)
Dissemination by a Northeast LIC
In-person dissemination of the study—DW meeting points in a large city in a Northeastern state
Support and dissemination by a group of LICs in the most populous state (SP)
Contact with a LIC in another capital of the Northeast, requesting support
In-person dissemination of the study—DW meeting points in this other capital of the Northeast
Contacts with new LICS in the Midwest states, requesting support

LIC: leaders or influencers in the category.

It took 45 shifts of in-person activities to disseminate the web survey, carried out during online data collection, totaling a workload of approximately 132 hours, at DW meeting points, such as squares, shopping mall parking lots, and public roads, in four cities: Salvador, Lauro de Freitas, and Feira de Santana, in the state of Bahia, and Recife, in the state of Pernambuco. This choice was not guided by the criteria of representativeness of these cities for the purposes of the research, but by their availability. On these occasions, conversations were held with the workers about the research and once the workers expressed interest in participating, their cell phone number was collected in order to send the survey link via WhatsApp. Subsequent calls were made reiterating that this was a non-profit public university research, and indicating that its origin could be verified on the Instagram profile.

Procedures to formalize the researchers’ access—legal letters—including to external areas of establishments, were necessary, given that the dissemination team was denied access to some of these places, such as shopping malls and supermarket parking lots, even though the team in the field was properly identified with the project’s T-shirt and badge.

It was found that sometimes the link to respond to the questionnaire was only enabled (“clickable”) if there was some communication between the receiver and the cell phone sending the link. The creation of an interactive card (“pdf” format) aimed to solve this problem.

The web survey was also disseminated in live broadcasts carried out by LIC on YouTube, as well as on radio, television and online newspapers, and the boosting feature was used in an Instagram publication in the last two weeks of data collection.

The figure shows the number of respondents weekly and over the course of the data collection, and it is possible to identify this growth from some strategies developed by the research team, with emphasis on in-person dissemination activities and contacts with LICs with relevant insertion in the category (Table 2). An increase in the response pattern in week 7 occurred when the research team managed to contact LICs with relevant insertion in social media and DW associations.

**Figure:**
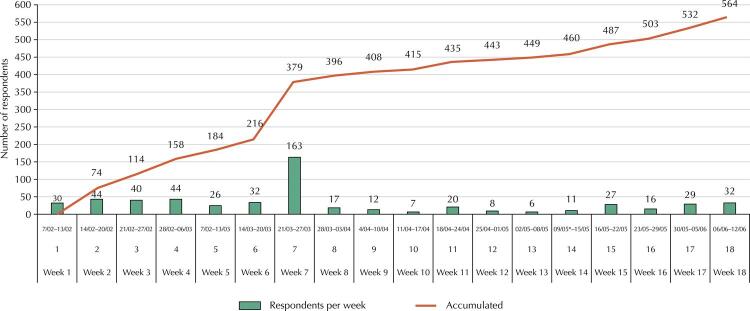
Cumulative frequency of participation of delivery workers in the EpisSAT Entregadores web survey over four months and each week of data collection.

## DISCUSSION

We believe that the strategies adopted for data collection with a hybrid dissemination work made this research possible. After four months applying the web survey, 564 delivery workers, 543 men and 18 women, responded to the online questionnaire. The questionnaire link was shared daily, and, at the same time, the web survey required in-person dissemination, which is why it was hybrid; interacting with DW; and articulating with the LICs.

Thus, the methodological path of this survey presented specificities, most of which were foreseen in the study planning, even when LICs admitted an insurmountable difficulty in terms of reaching more than 100 respondents. The difficulty for the DW to interrupt the “rush” (*correria*—a slang term used to refer to the arduous daily workday) to respond to a questionnaire was suggested in the study planning.

Abílio^19^ refers to DW who works on digital platforms as a “subordinate self-manager”: manager of themselves, but with clear subordination to the company, ready to answer the call at any time, but without remuneration for the waiting time, the “just-in-time worker”^3,19^. Additionally, the intense working hours, which exceeded the average of 75 effective weekly hours in October 2020, is what makes this category part of the social groups that are difficult to reach by epidemiological research^20^.

The unpaid time dedicated to the questionnaire was in dispute with the constant pressure of the workday, in which the DW remain alert to meet the next delivery request, in addition to the physical exhaustion, implying renunciation of any other activity ^19,20^. Study initiatives with worker populations living in precarious conditions, who are paid on demand, or hard-to-reach populations use a system to encourage participation^4,5,13,17^. In a Swedish study on precarious work, the respondent who indicated and assured the participation of two more respondents was rewarded financially or with a bonus: a compensation of approximately 11 dollars or a movie ticket^4^.

The survey dissemination via social media, especially messaging applications, still tends to generate distrust among recipients, who may ignore the content just because they are uncertain about the suitability of the message and the security of the link, which often is not even clicked. This required repeated communication with the DW about the validity of the link. Surveys conducted with the aid of a program, in the case of the Web-based Respondent Driven Sampling, with a record of the participant’s personal identity, generate a link for each respondent, which can establish greater security regarding the origin of the invitation^4,17^. However, other disadvantages may arise, especially in contexts where the need to identify the participant may imply refusal or information bias. This is, therefore, not always a valid measure for research in the world of work, in contexts where even recording the date of birth generates fear of being identified, as already mentioned. The control of the workforce, permanent surveillance, and blocking measures show the real modality of the delivery workers’ subordination relationships^8,19^.

The hybrid character of the study manifests itself especially with the in-person work of dissemination on the streets and overcoming obstacles in this territory, in addition to the online work. The difficulties of accessing the workers in person, to explain the research and convince them to participate, had a direct impact on obtaining responses to the questionnaire. The result of this web survey would not be achieved if only the link were sent to the target population by social media, as would be expected in a typical study of this nature^16^.

Considering the number of cell phones to which the survey link was sent, it is possible to admit that there was a low response to this isolated procedure. It was difficult to obtain the return of messages sent privately, which could be due to the delivery workers’ fear regarding the veracity of the study, as evidenced in some return messages from workers who pointed out, among other things, that: “this is fraud,” “that’s a game,” “it’s a well-made game,” “I’m not going to fall for this by clicking on the link.” As strategies adopted in real time by the team, a more dialogic and personal contact was sought, sending new messages with content that would help clarify doubts about the veracity of the survey.

It was observed that Instagram played a relevant role in encouraging continued adherence of LICs regarding the dissemination of the web survey, as occurred, for example, when their profiles were mentioned (“tagged”) in the web survey publications and they republished them. However, it is possible that the use of this social media by DW, despite being a young category, is less frequent than in other populations. It is important to highlight the high consumption of the Internet data package for using Instagram. In addition, it was found that the difficulty workers had in accessing the internet, evidenced during in-person activities, may have limited access to the questionnaire. This is an apparent paradox, given they are subordinate workers who work on digital platforms. Nonetheless, this is the digital divide arising from the cost of services.

These specificities highlight the methodological itinerary undertaken in this study. Comparing the procedures adopted in this web survey with other studies that used a program to recruit participants and collect data online, it is observed that this resource, not accessible to this study, establishes facilitators. The program sends e-mails or invitations with links, where each link is specific to a participant and associated with the respondent’s identity^4,17^. Despite being a promising resource, which has been used in Swedish initiatives with some success^4,5^, given the knowledge accumulated during this web survey on the characteristics that mark contemporary work and its high degree of precariousness, the exclusive virtual (online) way of carrying out research with DW may not present the feasibility observed in studies with formal categories, with full employment ties.

Expanding the dissemination of the web survey on radio and TV news was a challenge. Relying on the University’s own resources (communication consultancy), it was possible to reach a national TV news program^21^ with a large audience in social groups with low purchasing power and quality of life, where the DW come from, among other TV news. These initiatives aimed at providing an additional source of information for the DW about the origin of the research at the public university and the validity of the link provided in the project’s social media^22,23^.

Epidemiological studies with hard-to-reach populations face a major challenge, which is to access at least an acceptable number of participants. When successful, the study is of total relevance, given the scarcity of population studies with these individuals who constitute socially vulnerable groups. However, one of the major limitations of these studies is ensuring the representativeness of the target population, as researchers do not have the population data to plan and define the usual sampling strategy.

At EpisSAT Entregadores, although the web survey recorded 564 completed questionnaires, with the participation of DW from different states of the country, there was a wide variation in the proportion of responses, with the highest participation in the states of Bahia and Sao Paulo. In the first case, the good participation reflects the location of the research team and the viability of face-to-face dissemination activities in the capital of Bahia, and, in the second case, the high proportion, in addition to resulting from the greater number of workers operating in the capital of São Paulo, certainly reflects the greater presence of DW unions and associations in the country’s largest city.

A characteristic of this study, which dealt with challenges and created alternatives and initiatives that could innovate in the face of obstacles, was the lack of funding by any research agency. Thus, all the stages were carried out with strategies aimed at guaranteeing the validity of the study, adopting the perspective of the research subjects’ participation in all its stages. In this sense, despite the limits of financial resources, it was possible to meticulously plan the strategies and methodological development of the web survey.
